# Factors associated with women’s preference for an over-encapsulated Dual Prevention Pill: Findings from two clinical crossover trials among women in South Africa and Zimbabwe

**DOI:** 10.21203/rs.3.rs-6683281/v1

**Published:** 2025-06-09

**Authors:** Sanyukta Mathur, Marlena Plagianos, Barbara Friedland, Irene Bruce, Brady Burnett-Zieman, Adlight Dandazi, Petina Musara, Nkosiphile Ndlovu, Natasha Sedze, Siyanda Tenza, Krishnaveni Reddy, Shile Zulu, Lisa Haddad, Caroline Murombedzi, Nyaradzo Mgodi, Thesla Palanee-Philips

**Affiliations:** Population Council; Population Council; Population Council; Population Council; Population Council; University of Zimbabwe; University of Zimbabwe; University of the Witwatersrand; University of Zimbabwe; University of the Witwatersrand; University of the Witwatersrand; University of the Witwatersrand; Population Council; University of Zimbabwe; University of Zimbabwe; University of the Witwatersrand

**Keywords:** new technologies, multi-purpose prevention technologies, family planning, HIV prevention, South Africa, Zimbabwe, preference, dual prevention, prevention, mixed-methods

## Abstract

**Background:**

A dual prevention pill (DPP) for co-delivery of pregnancy and HIV prevention is currently in development and will offer an alternative to taking two separate products. We examined behavioral, partnership/interpersonal, social, and structural correlates of preferences for an over-encapsulated DPP (as a proxy for the product in development, consisting of oral contraceptive pill and oral PrEP pill) among women and adolescent girls in South Africa and Zimbabwe to inform DPP introduction and counseling strategies.

**Methods:**

This secondary analysis used data from two clinical crossover studies that compared the acceptability, adherence, and preference for the over-encapsulated DPP versus separate PrEP and oral contraceptives. HIV-uninfected, non-pregnant cisgender women were enrolled in Johannesburg, South Africa (n = 96, ages 18–40) and Harare, Zimbabwe (n = 30, ages 16—24). Participants were randomized 1:1 to use either DPP or two separate pills for three 28-day cycles, then switched. Preferences were measured at the end of crossover and via exit in-depth interviews.

**Results:**

The mean age was 27 years in South Africa and 19 years for Zimbabwe. Most participants had completed secondary school. Childbearing was more common in Zimbabwe (97%) than South Africa (74%). Pregnancy prevention was highly valued in both sites (~ 90%), but Zimbabwean participants voiced greater concern about HIV. DPP preference was higher in Zimbabwe (62%) than South African (39%). In South Africa, DPP preference was associated with behavioral factors (anal sex, experience of sexual violence, relationship conflict, and transactional relationships/sex) and product characteristics (ease of use, side effects). In Zimbabwe, concern about HIV acquisition, interpersonal power, and structural factors (i.e., food insecurity and housing security) were associated with DPP preference. Qualitative interviews highlighted the DPP’s simplicity as key advantage over two separate pills, though participants desired a smaller DPP and more discreet packaging to reduced stigma and ease use.

**Conclusions:**

While women in both countries value integrated HIV and pregnancy prevention, distinct demographic, behavioral, and social factors influence DPP preference. Tailoring DPP introduction and support strategies to address specific challenges–such as relationship dynamics and product usability–may optimize the acceptance and effectiveness of dual prevention strategies.

## INTRODUCTION

Women of reproductive age have overlapping needs for both HIV and pregnancy prevention. In 2023, an estimated 1.3 million people globally were newly infected with HIV; 44% of those were women and girls. (1) In sub-Saharan Africa, women and girls accounted for 62% of all new HIV infections.(1) At the same time, recent analysis show that rates of unintended pregnancy are on average greatest in sub-Saharan Africa compared with the other regions globally.(2) A single product that simultaneously prevents pregnancy and HIV - a multi-purpose prevention technology (MPT) - could meet these dual needs and ease the use and adherence burden on women.

Prior research on MPTs highlights that women are interested in and value novel products offering protection against multiple health risks, such as unplanned pregnancy, HIV, and other STIs in a single solution.(3–7) While condoms offer multi-purpose protection, their effective use has been hampered by cultural perceptions around condom use and the dynamics of sexual negotiation/ reliance on male partners for consent to use.(8) Studies also show that preference can vary by a host of socio-behavioral factors (e.g., partner perspectives) and product attributes (e.g., administration, regimen, etc.).(6) Additionally, although preference for MPTs can vary by context (4) few studies present cross-country comparisons. Since most MPTs are in pre-clinical development, much of the MPT preference research to-date has relied on placebo products or survey research with hypothetical product descriptions.(6) Product preferences may change once women have a chance to use MPTs with intended therapeutic activity.

The dual prevention pill (DPP), a daily oral pill co-formulated with the ingredients in oral HIV pre-exposure prophylaxis (oral PrEP) and a combined oral contraceptive (COC), will be the first novel MPT to be introduced for dual HIV and pregnancy prevention.(9) To begin to address the above mentioned knowledge gaps for this product, we evaluated the preference, acceptability and adherence of an over - encapsulated DPP (containing a COC pill and an oral PrEP pill as a proxy for the co-formulated product in development) versus PrEP and COCs used separately (2-pill regimen [2PR]) via two clinical crossover studies in South Africa and Zimbabwe.(10) According to 2023 estimates, HIV incidence in South Africa was 4.55 per 1000 population with 89,000 women aged 15 and over newly infected with HIV.(11) HIV incidence in Zimbabwe was 1.48 per 1000 population with 7500 new infections among women aged 15 and older.(12) Unintended pregnancy burden remains high among women aged 15–49 years in both countries, at 81 and 74 per 1000 women in South Africa and Zimbabwe respectively.(13) Additionally, in Zimbabwe, the adolescent birth rate is high at 108/1000 girls aged 15–19 years, whereas the rate is 41/1000 girls in South Africa.(13) These statistics underscore the potential for the novel DPP to meet adolescent girls and women’s dual protection needs. In this secondary analysis, we sought to understand the factors associated with preference for the DPP after women had used both an over-encapsulated DPP and the 2PR regimen in both studies.

Our analysis is based on a comprehensive framework for DPP introduction which considers how individual-level, provider-level, and product-related factors may influence women’s intention for product use and, ultimately actual use of the product.(14) Therefore, in this analysis, we explore if prevention motivation, individual behaviors (such as number of partners, frequency of sex), interpersonal/partnership characteristics (such as experience of sexual violence, relationship control in intimate partnerships, concurrency), and social and structural factors (such as anticipated stigma, food insecurity), as well as product attributes (such as ease of use, side effects) are associated with preferences for a DPP. Additionally, we add qualitative insights on participant reflections about the DPP and their product preferences.

## METHODS

### Study Setting

The clinical crossover studies were conducted at two sites: the Wits RHI Research Centre Clinical Research Site (CRS) in Hillbrow, Johannesburg, South Africa, and the Zengeza CRS in Chitungwiza, Zimbabwe.

### Study Population

Clinical trial participants included sexually active women aged 18–40 years in South Africa and 16–24 years in Zimbabwe, recruited from family planning (FP), HIV, and sexual and reproductive health (SRH) clinics, as well as the general population. The trials enrolled women who were already using oral contraceptive pills for at least three months to leverage familiarity with daily pill-taking and associated side effects. Prior to enrolment, participants were assessed for levels of comfort taking a proxy for the study product. This involved clinician-observed swallowing of a large Vitamin C capsule, similar in size to the study product, during Screening.

### Study Design and Randomization

Trial participants were randomized into two sequences for a six-month crossover trial. Sequence 1 participants used a single daily DPP for three consecutive 28-day cycles followed by the 2PR (oral PrEP and COCs used separately) for three cycles. Sequence 2 participants followed the opposite order. In South Africa, participants had the option to continue onto a choice period, where they could use the regimen of their choice. Exit interviews were conducted with study participants after the crossover. Trial design and procedures are described more fully elsewhere.(10)

### Data Collection

At enrollment and each monthly study visit, participants completed a behavioral questionnaire via computer-assisted self-interview (CASI) in English or the local language–isiZulu in South Africa and Shona in Zimbabwe. Surveys assessed background characteristics (baseline); product adherence and sexual behaviors (monthly), and product preference and acceptability (end of treatment regimen). Each session lasted approximately 30 minutes and was conducted privately on handheld tablet devices, with study staff on hand to address technical challenges.

Qualitative exit in-depth interviews (IDIs) were conducted using a standardized IRB approved guide to further explore product preferences, as well as gain insights on the eventual co-formulated DPP tablet (participants were shown images of the pill and packaging). In South Africa, participants were purposively sampled among those who chose the DPP or 2PR for the choice period of the trial. Interviews, conducted in English or the local language per participant preference, lasted 40–60 minutes, were audio recorded, transcribed, and translated into English, when relevant. In Zimbabwe, all study participants were interviewed. Data collection began in August 2022 and was completed in January 2024.

### Data Analysis

Preference for the DPP versus the 2PR was measured quantitatively by the proportion of participants expressing a preference for each regimen at the end of the crossover period. We summarized preferences and responses to key socio-demographic, attitudinal, behavioral, partnership/interpersonal, and social and structural factors; countries were analyzed separately for associations with preference. Associations between participants’ baseline characteristics with endline regimen preferences, as well as product attributes and regimen preference at endline were assessed with Fisher’s exact test for categorical variables and t-test or Wilcoxon test for continuous variables that were normally or not normally distributed, respectively. Items that were significantly associated with preference at the 0.20 level in bivariate analyses were included in multivariable logistic regression modeling probability of preferring the DPP. We used backward elimination to obtain a final model, keeping factors that were significant at the 0.05 level. As a supplementary analysis, we combined the data from both countries and ran bivariate and multivariate models using forward selection to determine a parsimonious model.

We assessed socio-demographic variables (e.g., age, education, childbearing experience), interest in HIV and pregnancy prevention (e.g. importance of pregnancy prevention), sexual behaviors (e.g. multiple partnerships, anal sex), partner characteristics and dynamics (e.g. partner type, sexual violence experience, interpersonal power), and social/structural factors (e.g. going whole day/night without eating, having a place to stay, anticipate negative experiences with DPP use). Product attribute variables included ease of use of the daily regimen, size, side effects, and impact on sex.

IDI data were analyzed using a simplified rapid analysis technique, creating summary tables by themes and by participants to facilitate cross-participant and cross-theme analysis.(15)

### Ethics

The clinical study protocols, consent forms, recruitment materials, and data collection instruments were approved by the Population Council Institutional Review Board (New York, USA). In South Africa, the study was approved by the University of the Witwatersrand Human Research Ethics Committee and South African Health Products Regulatory Authority. In Zimbabwe, the study was approved by the Medical Research Council of Zimbabwe, the Medicines Control Authority of Zimbabwe, the Joint Research Ethics Committee of the University of Zimbabwe, the Ministry of Health and Child Care of Zimbabwe, the Chitungwiza City Health Ethics Committee, and the Research Council of Zimbabwe. Both studies adhered to international guidelines, including the ICH-GCP E6 (R2) standard, U.S. federal regulations, and standard operating procedures at the clinical trial sites and the Population Council. All participants provided written informed consent prior to undergoing any study procedures and were compensated in accordance with local ethical standards. The trials were registered on ClinicalTrials.gov (NCT04778527-South Africa and NCT04778514-Zimbabwe).

## RESULTS

### Participant Characteristics

A total of 85/96 women in South Africa completed the crossover portion of the study (mean age, 27 years), 21 of whom participated in an IDI. In Zimbabwe, 26/30 completed the study (mean age, 19 years), all of whom participated in an IDI (Table 1). Most participants in South Africa had never been married (95%) whereas most participants in Zimbabwe were currently married (47%) or divorced/widowed (30%). Majority of the participants in both countries had completed secondary school (81% in South Africa and 97% in Zimbabwe). Nearly a third of the South African participants were currently in school (tertiary) whereas only one Zimbabwean participant was in school. Childbearing experience was high in South Africa (74%) and nearly universal in Zimbabwe (97%).

Most participants in both study contexts noted that pregnancy avoidance was very important to them (89% and 90%, South Africa and Zimbabwe respectively), whereas concern about acquiring HIV was slightly different– in South Africa 44% were very worried and 13% somewhat worried about acquiring HIV compared to 57% and 27% of participants in Zimbabwe.

Behavioral characteristics of participants were slightly different in the two study contexts. South African participants reported 3 recent sexual partners on average whereas participants in Zimbabwe reported 2 sexual partners on average; 12% in South Africa reported no vaginal sex between screening and enrollment compared to 24% in Zimbabwe. Similar proportions in both sites reported no condom use at last sex (61% in South Africa, and 64% in Zimbabwe). Anal sex was reported by 13% of South African and 7% of Zimbabwean participants.

Interpersonal/partnership characteristics in the two study contexts also differed somewhat; 86% of South African participants noted their main partner was a boyfriend, whereas 54% of Zimbabwean participants’ main partner was their husband. Most participants in both contexts reported that their main partner was HIV negative (63% in South Africa, 57% in Zimbabwe). When asked about partners’ concurrent partners, 14% in South Africa reported that their partner did not have other partners whereas most participants in Zimbabwe (93%) either thought their partner had other partners or reported they did not know. Experience of sexual violence was similar in both contexts with 18% in South Africa and 17% in Zimbabwe reporting ever experiencing sexual violence. Using the relationship control sub-scale of the sexual relationship power scale [14], participants in South Africa reported more control in their sexual relationships than participants in Zimbabwe (2.19 average scale score vs. 2.44; higher average score implies less control). Engagement in transactional relationships was more common in Zimbabwe (38%) than South Africa (26%).

Social and structural challenges were also observed among study participants in both countries. Food insecurity (going a whole day/night without food in the past 4 weeks) was reported by 50% of the participants in South Africa versus 60% of Zimbabwean participants. Housing insecurity (not having a place to stay or store their belongings) was reported by 40% of participants in South Africa and 47% in Zimbabwe. More participants in South Africa reported facing negative attitudes in the due to their contraceptive pill use (15%) than participants in Zimbabwe (7%). Anticipated stigma associated with DPP use was also reported by more participants in South Africa (51%) than Zimbabwe (33%).

### DPP Preference and Product Attributes

At the end of the crossover period (Table 2), a minority of participants in South Africa preferred the DPP versus the 2PR (39%; p = 0.03) while a majority preferred the DPP in Zimbabwe (62%; p = 0.24). After using each regimen during the crossover period, participants were asked to rate specific product characteristics; 61% of South African participants found it easy to take the DPP every day versus 83% in Zimbabwe. In South Africa 11% of participants reported that the size of the DPP was too big, and they could not swallow it, whereas none of the participants in Zimbabwe said the same. Side effect experiences were also different; approximately 14% of participants in South Africa noted that side effects bothered them a lot whereas none of the Zimbabwean participants reported the same. Finally, 49% of the participants in South Africa thought that using the DPP improved their sex life, 86% did so in Zimbabwe.

### Factors associated with DPP Preference

Table 3 presents bivariate associations between various factors and preference for the DPP regimen at the end of the crossover period for each country.

In South Africa, 77.4% of the participants who preferred the DPP had two or more partners in the past 3 months versus those who preferred the 2PR (51.0%, p = 0.021). 12.9% of the participants who had engaged in anal sex preferred the DPP compared to 26.5% who preferred the 2PR (p = 0.172). DPP preference was higher among participants who had ever experienced sexual violence (33.3%) versus the 2PR (12.5%, p = 0.042). Likewise, participants who reported less control in their relationships preferred the DPP compared to participants who reported more control in their relationships (p = 0.026). DPP preference was higher among women who had engaged in transactional sex in the past 3 months versus the 2PR (40.6% vs. 21.6%, p = 0.083). Additionally in South Africa, several product characteristics were associated with DPP preference. DPP preference was higher among participants who found the regimen easy to use versus the 2PR (73.1% and 51.4% respectively, p = 0.076). If participants did not have side effects, they preferred the DPP, however if the side effects bothered them a lot, they preferred the 2PR (p = 0.064).

In Zimbabwe, among participants who preferred the DPP 37.5% were very worried about HIV acquisition, whereas 90.0% of the participants who preferred the 2PR were very worried about HIV acquisition (p = 0.037). Among participants who preferred the DPP 78.6% noted that their partner had more power in their relationship compared to 44.4% of the participants who preferred the 2PR (p = 0.041). Among participants who preferred the DPP 25.0% had never experienced food insecurity compared to 70.0% of participants who preferred the 2PR (p = 0.095). 6.3% of the participants who preferred the DPP never had a regular place to stay/store things whereas 30% of the participants who preferred the 2PR noted the same (p = 0.089). Finally, most participants who preferred the DPP thought the DPP regimen was easy (93.3%) versus 62.5% of the participants who preferred the 2PR (p ≤ 0.032)

In South Africa, only two factors remained significantly associated with DPP preference in the multivariable model: no anal sex (adjusted OR:4.01,95% CI: 1.039–15.862) and lower relationship control (adjusted OR: 3.35, 95% CI: 1.268–8.852). We were unable to assess likelihood of DPP preference in Zimbabwe with a multivariate model due to overfitting with the small sample size. In the bivariate model combining data from both countries, DPP preference was associated (at the 0.20 level of significance) with anal sex, number of partners, type of partner (boyfriend, husband, other), interpersonal power, having had transactional sex, food or housing insecurity, and intimate partner violence. However, in the multivariable model only lower relationship control was significantly associated with DPP preference (OR: 3.15, 95% CI: 1.378–7.212).

### Qualitative Insights on Product Preference

In South Africa, 21 women participated in exit IDIs, one who withdrew after the crossover period and 20 who completed the choice period (10 chose DPP, 10 chose 2PR). In Zimbabwe, all 26 participants who completed the study and one who withdrew early participated in the exit IDIs.

In Zimbabwe, there was a clear preference for the DPP, both in the qualitative and the quantitative data. In South Africa, two-thirds (14/21) of the IDI participants said they preferred the DPP at study exit even though a majority reported preferring the 2PR after the crossover period (in the quantitative data) and more women chose the 2PR for the choice period. Women’s reasons for preferring the DPP were similar across the two countries.

The main reason for preferring the DPP was the simplicity of taking a single pill versus two separate pills, which made it easier for adherence.

Because it does two jobs at once, I don’t have to use multiple pills. If it was available at my nearest clinic, I would not be hesitant to collect it...(South African participant, 31-year-old)

It really helped me a lot because I will be just taking one pill. I do not have to take two separate pills; I will be just taking one which I will know that it is preventing both [HIV and pregnancy]. (Zimbabwean study participant, 24-year-old)

Several South African participants noted that although they preferred the DPP, they had chosen the 2PR for the choice period because of the over-encapsulated DPP’s bulky packaging.

Because the containers [for COCs and PrEP] are portable, you can place them under your clothes, in your closet or anywhere in your small space. Unlike the DPP package, I had to put them where my shoes are so that people won’t be asking too much about them... Just change the packaging. The DPP it’s not difficult to drink every day since it’s just one pill. (South African participant, 30-year-old)

Despite the bulky packaging, some participants mentioned that there was less chance of stigma with the DPP because the pill and the packaging did not look like HIV medication.

I think one DPP will be easy because you don’t have to hide so the partner will think it is only for prevention unlike the two separate pills where the partner might think you are HIV [sic, person living with HIV] as you are taking more than one pill. (South African participant, 28-year-old)

De-linking the look and packaging of DPP from HIV treatment medication is important. One young woman in Zimbabwe reported inadvertent disclosure of study participation when a friend saw her with the 2PR.

The DPP were okay to store them in the house because they were in a plastic [blister] pack. One would think maybe they are just painkillers unlike the two separate pills which were in containers.. One day my friend came into my house, and I had not put them [PrEP pills] back where I keep them... She was shocked and said, “Ah, you are now drinking ARVs?” And I explained to her,.., she was not convinced. That is when I had to show her the study visit card. (Zimbabwean participant, 17-year-old)

**Figure 1:**
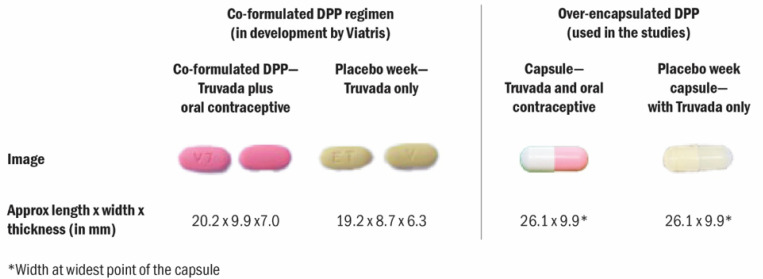
Images and dimensions of the co-formulated DPP regimen currently in development and the over-encapsulated DPP used in the studies

When participants were shown images of the co-formulated DPP that is currently under development (Fig. 1), they approved of the smaller size and narrower shape compared to the over-encapsulated DPP they had used in the trials. One South African participant, for instance, noted that while she had chosen the 2PR during the choice period of the trial, she would prefer the DPP. She had found the over-encapsulated DPP harder to swallow than PrEP and thought the slightly smaller size of the eventual DPP product will be easier to use. Participants also noted that the color (pinkish hue of the DPP tablet) was pleasing and again emphasized preference for the product to be distinct in look and packaging from HIV treatment.

## DISCUSSION

The study explored preference for the DPP versus a 2-pill regimen using data from two clinical crossover studies with women in South Africa and Zimbabwe, revealing notable differences in demographics, behaviors, and product preferences. These are the first studies to explore preference of an MPT compared to single indication products after actual use versus prior studies testing hypothetical MPT interest based on placebo products.

Product preference differed by country context-with South African participants preferring the 2-pill regimen and Zimbabwean participants preferring the over-encapsulated DPP. Prior MPT research has found country-level differences in MPT product preferences, potentially due to familiarity with the product regimen.(4, 6) It is possible that in Zimbabwe, where oral contraceptives are a prominent method for pregnancy prevention, young women who were part of the study might have been more willing to consider a daily oral MPT similar to oral contraceptives.

Factors associated with product preference also seemed to differ slightly in the two studies. In South Africa, several behavioral factors (anal sex, experience of sexual violence, relationship conflict, and transactional relationships/sex) and product characteristics (ease of use, side effects) were significantly associated with DPP preference. In Zimbabwe, concern about HIV acquisition, interpersonal power, and structural factors (i.e., food insecurity and housing security) were associated with product preferences. Site level differences could be due to the socio-demographic and behavioral differences in the study cohorts. It is notable however, that in both sites partner dynamics – relationship conflict, sexual violence, or interpersonal power– were associated with a preference for the DPP; signaling DPP as a potentially desirable option for women who are in challenging partnerships. Prior research into contraceptive and PrEP preferences has pointed to women’s empowerment and partner/interpersonal dynamics as key influencers of product preference. For instance, Kusemererwa et al., found that women at risk of HIV preferred contraceptive methods that allowed for privacy and independence from their partners.(16) Rousseau et al. found that lower relationship power among women was associated with reduced PrEP interest and uptake, particularly in contexts where women were in relationships with higher-risk partners. (17) Qualitative formative research in our study sites with women and healthcare providers also found strong interest in the DPP, as a way to enhance self-care and control women’s own prevention needs.(18, 19) Our analysis speaks to the importance of having a menu of prevention options that participants can choose based on personal preferences and circumstances.

Our data reveals that product characteristics are key for new product preferences. Study participants, especially in South Africa, had trouble with the large size of the over-encapsulated DPP used in the study. The eventual co-formulated product will be smaller in size. Additionally, the packaging of the over-encapsulated DPP used in the study might have led some participants to note a preference for the 2PR over the DPP during these studies. A few studies in the region that have examined the design and packaging preferences for MPTs, found preference for discreet, small, and non-medical packaging.(20, 21) Indeed women in our study preferred the images of the co-formulated DPP in terms of its size and look. For both the over-encapsulated product used in our study and the eventual DPP product, participants appreciated that it looked different from HIV treatment medications which are often well-recognized and often stigmatized. Additional research in the region on preferences for different PrEP delivery methods reflect women’s desire for discreetness and control over their health.(22, 23) This preference can be influenced by the nature of their relationships and the perceived risks associated with discussing PrEP with partners or others.(24) Packing and marketing of the DPP and future MPTs should aim towards compact and non-medical packaging to support product uptake and use.

Our analysis has several limitations. We were underpowered to fully understand the characteristics associated with product preference, particularly in Zimbabwe. Larger studies in different contexts (in SSA and other regions of the world) are needed to understand DPP acceptability and preference. The qualitative data did not explore, and the participants did not reveal if other issues like food or housing insecurity influenced their preference or experience with the study products. The qualitative data also did not examine how interpersonal relationship dynamics might have changed over time to influence product preference. These issues warrant further examination for DPP introduction and to counsel clients on effective use. Other analyses from these study data show high acceptability for the DPP but low adherence to either study regimen; subsequent analysis may need to explore how preference and adherence are related for the DPP or other MPT products.

There was an interest in novel prevention products and the DPP specifically (though maybe not the particular product used in these studies). We also found that DPP preference might differ by context and partnership characteristics. Our mixed methods approach also highlights how product preference was shaped by product characteristics and implications for storage, portability, and use. Understanding DPP preference and the behavioral, social, and structural factors, and product attributes that are associated with DPP preference can inform plans for DPP introduction, counseling, and support for effective use.

## Figures and Tables

**Table 1:** Baseline respondent characteristics by country

	South Africa		Zimbabwe	
	N = 96		N = 30	
**Socio-demographic characteristics**				
Age [mean (range) in years]	27.4 (18.0–40.0)		19.4 (16.0–24.0)	
	n	%	n	%
Marital status				
Never married	91	94.8	6	20.0
Married	2	2.1	14	46.7
Divorced/Widowed	2	2.1	9	30.0
Other	1	1.0	1	3.3
Currently in school	31	32.3	1	3.3
Highest level of schooling completed				
Primary	2	2.1	1	3.3
Secondary	78	81.3	29	96.7
Beyond secondary	16	16.7	0	0.0
Childbearing experience (ever)	71	74.0	29	96.7
**Interest in Pregnancy and HIV prevention**				
Importance of avoiding pregnancy				
Very important	86	89.6	27	90.0
Somewhat important	3	3.1	1	3.3
A little important	0	0.0	1	3.3
Not important	5	5.2	0	0.0
Don’t know	2	2.1	1	3.3
Worry about HIV acquisition in the next 3 months				
Very worried	42	43.8	17	56.7
Somewhat worried	12	12.5	8	26.7
Not very worried	18	18.8	0	0.0
Not worried at all	24	25.0	4	13.3
Refused	0	0.0	1	3.3
**Behavioral factors**				
Number of sex partners in the past 3 months (Mean (SD, range))	3.12	3.1 (4.3, 0–30)	1.97 (1.99, 0–10)	
0/1				
2+				
		%		%
Vaginal sex since last visit *				
No vaginal sex	11	12.0	7	24.1
Once or twice	37	40.2	10	34.5
3–4 times	27	29.4	4	13.8
5–10 times	4	4.4	3	10.3
Daily or almost daily	12	13.0	4	13.8
Refused	1	1.1	1	3.5
Condom use at last act of vaginal sex (of those who had vaginal sex)				
No	49	60.5	14	63.6
Yes	31	38.3	8	36.4
Refused	1	1.2	0	0.0
Anal sex since last visit*				
No anal sex	73	79.4	27	93.1
Once or more	18	13.0	2	6.9
refused	1	1.1	0	0.0
**Interpersonal/Partnership characteristics**				
Main partner type				
Boyfriend	78	85.7	9	32.1
Husband	77	7.7	15	53.6
other	6	1.1	4	14.3
Partner(s) living with HIV or their HIV status unknown				
HIV-negative	60	62.5	17	56.7
HIV-positive/Don’t Know status	30	31.3	11	36.7
Refused/missing	6	6.3	2	6.7
Partner(s) has other partners				
Yes/Don’t know	77	80.2	28	93.3
No	13	13.5	0	0.0
refused/missing	6	6.3	2	6.7
Sexual violence experience				
Never	73	76.0	22	73.3
Ever	17	17.7	5	16.7
refused/missing	6	6.3	3	10.0
Relationship control scale score (Higher score = less control, Mean (SD, range))	2.19 (0.58, 1.13–3.50)		2.44 (0.52, 1.50–3.38)	
In the past 3 months, engaged in sex or started/stayed in relationship for financial or material gain				
No	71	74.0	19	63.3
Yes	25	26.0	11	36.7
**Social and Structural Characteristics**				
Went a whole day and night without eating due to a lack of food in the past 4 weeks				
Never	48	50.0	12	40.0
Rarely	13	13.5	7	23.3
Sometimes	31	32.3	5	16.7
Often	4	4.2	6	20.0
Had a place to stay or store your things regularly in the past 4 weeks				
Never	25	26.0	6	20.0
Rarely	3	3.1	6	20.0
Sometimes	10	10.4	2	6.7
Often	58	60.4	16	53.3
Experienced negative attitudes or behaviors due to use of contraceptive pills	14	14.6	2	6.7
Anticipate negative experiences from partners, family, peers or community for using DPP	49	51.0	10	33.3

**Table 2:** Regimen preference and perspectives on product characteristics by country

*DPP Product characteristics (endline)*	South Africa		Zimbabwe	
	*(n = 66)*		*(n = 26)*	
	n	%	n	%
Product preference				
DPP	32	38.6	16	61.5
2PR	51	61.5	10	38.5
How easy was it to take the DPP every day?				(n = 24)
Very Easy	40	60.6	20	83.3
Somewhat Easy	18	27.3	0	0.0
Somewhat Difficult	6	9.1	3	12.5
Very Difficult	2	3.0	1	4.2
What is your opinion of the size of the regimen?				(n = 23)
Too big, I could not swallow it	7	10.6	0	0.0
Big, but I was able to swallow it	39	59.1	20	87.0
The size was alright, I had no trouble swallowing it	19	28.8	3	13.0
How much did side effects bother you?				(n = 23)
I did not have side effects	19	28.8	17	73.9
Side effects did not bother me	8	12.1	3	13.0
Side effects bothered me a little	30	45.5	3	13.0
Side effects bothered me a lot	9	13.6	0	0.0
How would you say that using the regimen affected your sex life?				(n = 21)
Improved my sex life a lot	32	48.5	18	85.7
Improved my sex life a little	5	7.6	15	23.8
Had no effect on my sex life	27	40.9	3	66.7
Made my sex life a little worse	1	1.5	0	0.0
Made my sex life a lot worse	0	0.0	0	0.0

**Table 3:** Bivariate associations between regimen preference and individual, behavioral, interpersonal, social and structural, and product characteristics, by country

	South Africa			Zimbabwe		
	% who preferred DPP (n = 32)	% who preferred 2PR (n = 51)	P-value	% who preferred DPP (n = 16)	% who preferred 2PR (n = 10)	P-value
**Socio-demographic characteristics**						
Age [mean (range) in years]	27.9	27.2	0.590	19.4	19.9	0.635
16–24 years	31.3	35.3		68.2	31.8	
25 + years	68.8	64.7		--	--	
Marital status			0.680			1.000
Never married	93.8	96.1		25.0	20.0	
Married	3.1	2.0		50.0	50.0	
Divorced/Widowed	0.0	2.0		18.8	30.0	
Other	3.1	0.0		6.3	0.0	
Currently in school			0.478			1.000
No	62.5	70.6		93.8	100.0	
Yes	37.5	29.1		6.25	0.0	
Highest level of schooling completed			0.0105			1.0
Primary	6.3	0.0		6.3	0.0	
Secondary	65.6	90.2		93.8	10.0	
Tertiary	28.1	9.8		0.0	0.0	
Childbearing experience	18.8	31.4	0.307	6.3	0.0	1.000
Nulliparous	81.3	68.6		93.8	100.0	
Parous						
**Interest in Pregnancy and HIV prevention**						
Importance of avoiding pregnancy			0.922			0.323
Very important	87.6	90.2		93.8	80.0	
Somewhat important	3.1	3.9		0.0	10.0	
A little important	0.0	0.0		0.0	10.0	
Not important	6.3	3.9		0.0	0.0	
Don’t know	3.1	2.0		6.3	0.0	
Worry about HIV acquisition in the next 3 months			1.000			**0.037**
Very worried	40.6	41.2		**37.5**	**90.0**	
Somewhat worried	15.6	13.7		**37.5**	**10.0**	
Not very worried	18.8	19.6		**0.0**	**0.0**	
Not worried at all	25.0	25.5		**25.0**	**0.0**	
**Behavioral Factors**						
Number of sex partners in the past 3 months	**3.8**	**2.8**	**0.108**	1.73	1.9	0.256
0–1	**22.6**	**49.0**	**0.021**	60.0	80	0.418
2+	**77.4**	**51.0**		40.0	20.0	
Vaginal sex since last visit (within the last 1–2 months?)			0.752			0.312
No vaginal sex	9.7	12.2		13.3	30.0	
Once or twice	48.4	32.7		46.7	30.0	
3–4 times	25.8	34.7		20.0	0.0	
5–10 times	3.2	6.1		13.3	10.0	
Daily or almost daily	12.9	14.3		6.7	30.0	
Condom use at last act of vaginal sex (of those who had vaginal sex)			0.227			1.000
No	50.0	65.1		69.2	71.4	
Yes	50.0	34.9		30.8	28.6	
Anal sex since last visit (within the last 1–2 months)			**0.172**			0.650
No anal sex	**87.1**	**73.5**		93.3	90.0	
Once or more	**12.9**	**26.5**		6.7	10.0	
**Interpersonal/Partnership characteristics**						
Main partner type			0.342			0.358
Boyfriend	80.7	91.7		40.0	22.2	
Husband	12.9	4.2		60.0	66.7	
Other	3.5	4.2		0.0	11.1	
Partner(s) living with HIV or their HIV status unknown			0.807			0.679
HIV-negative	64.5	68.8		66.7	55.6	
HIV-positive/Don’t Know status	35.5	31.3		33.3	44.4	
Partner(s) has other partners			0.588			1.000
No	9.4	15.7		0.0	0.0	
Yes/Don’t know	87.5	78.4		94.0	90.0	
refused/missing	3.1	5.9		6.3	10.0	
Sexual violence experience			**0.042**			1.000
Never	**66.7**	**87.5**		85.7	77.8	
Ever	**33.3**	**12.5**		14.3	22.2	
Who has more power in the relationship?			0.345			**0.041**
Partner	19.4	8.3		**78.6**	**44.4**	
Both equally	77.4	89.6		**7.1**	**55.6**	
Respondent	3.2	2.1		**14.3**	**0.0**	
Relationship control scale score (Higher score = less control)	**2.4**	**2.1**	**0.026**	2.5	2.3	0.469
In the past 3 months, engaged in sex or started/stayed in relationship for financial or material gain			**0.083**			1.000
No	**59.4**	**78.4**		68.8	70.0	
Yes	**40.6**	**21.6**		31.3	30.0	
**Social and Structural Characteristics**						
Went a whole day and night without eating due to a lack of food in the past 4 weeks			0.403			**0.095**
Never	40.6	54.9		**25.0**	**70.0**	
Rarely	9.4	13.7		**31.3**	**10.0**	
Sometimes	43.8	27.5		**25.0**	**0.0**	
Often	6.3	3.9		**18.8**	**20.0**	
Had a place to stay or store your things regularly in the past 4 weeks			0.843			**0.089**
Never	21.9	25.5		**6.3**	**30.0**	
Rarely	3.1	3.9		**37.5**	**0.0**	
Sometimes	6.3	11.8		**6.3**	**10.0**	
Often	68.8	58.8		**50.0**	**60.0**	
Experienced negative attitudes or behaviors due to use of contraceptive pills			0.551			0.508
No	81.3	86.3		87.5	100.0	
Yes	18.8	13.7		12.5	0.0	
Anticipate negative experiences from partners, family, peers or community for using DPP	2.6	2.7	0.483	2.9	2.8	0.394
**DPP Product characteristics**						
How easy was it to take the DPP every day?			**0.076**			**0.0316**
Very Easy	**73.1**	**51.4**		**93.3**	**62.5**	
Somewhat Easy	**26.9**	**28.6**		**0.0**	**0.0**	
Somewhat Difficult	**0.0**	**14.3**		**0.0**	**37.5**	
Very Difficult	**0.0**	**5.7**		**6.7**	**0.0**	
What is your opinion of the size of the regimen?			0.472			1.000
Too big, I could not swallow it	4.0	14.3		0.0	0.0	
Big, but I was able to swallow it	60.0	57.1		86.7	85.7	
The size was alright, I had no trouble swallowing it	36.0	28.6		13.3	14.3	
How much did side effects bother you?						1.000
I did not have side effects	**38.5**	**20.0**	**0.064**	73.3	75.0	
Side effects did not bother me	**3.9**	**17.1**		13.3	12.5	
Side effects bothered me a little	**53.9**	**42.9**		13.3	12.0	
Side effects bothered me a lot	**3.9**	**20.0**		0.0	0.0	
How would you say that using the regimen affected your sex life?			0.853			0.430
Improved my sex life a lot	42.3	51.4		86.7	66.7	
Improved my sex life a little	7.7	8.6		0.0	16.7	
Had no effect on my sex life	50.0	40.0		13.3	16.7	
Made my sex life a little worse	0.0	0.0		0.0	0.0	
Made my sex life a lot worse	0.0	0.0		0.0	0.0	

## Data Availability

The datasets used and/or analysed during the current study are available from the corresponding author on reasonable request.
